# Peroxisome Proliferator-Activated Receptors in Lung Cancer

**DOI:** 10.1155/2007/90289

**Published:** 2007-11-25

**Authors:** Venkateshwar G. Keshamouni, ShouWei Han, Jesse Roman

**Affiliations:** ^1^Division of Pulmonary and Critical Care Medicine, University of Michigan, Ann Arbor, MI 48109, USA; ^2^Division of Pulmonary, Allergy and Critical Care Medicine, Emory University, School of Medicine, Atlanta, GA 30322, USA; ^3^Atlanta Veterans Affairs Medical Center, Atlanta, GA 30033, USA

## Abstract

Peroxisome proliferator-activated receptors (PPARs) are ligand-activated transcription factors belonging to the nuclear hormone receptor superfamily. Their discovery in the 1990s provided insights into the cellular mechanisms involved in the control of energy homeostasis; the regulation of cell differentiation, proliferation, and apoptosis; and the modulation of important biological and pathological processes related to inflammation, among others. Since then, PPARs have become an exciting therapeutic target for several diseases. PPARs are expressed by many tumors including lung carcinoma cells, and their function has been linked to the process of carcinogenesis in lung. Consequently, intense research is being conducted in this area with the hope of discovering new PPAR-related therapeutic targets for the treatment of lung cancer. This review summarizes the research being conducted in this area and focuses on the mechanisms by which PPARs are believed to affect lung tumor cell biology.

## 1. INTRODUCTION

Lung cancer is the leading cause of cancer death in the world for both men and women [1]. Primary malignant cancers of the lung are classified into small cell lung cancer (SCLC) and nonsmall cell lung cancer (NSCLC) [2]. NSCLC accounts for 75% and SCLC constitutes the remainder. Based on the cellular phenotype, NSCLC is further subdivided into squamous cell carcinoma, adenocarcinoma, and large cell carcinomas [2]. Despite advances in understanding the mechanisms involved in carcinogenesis, the development of new surgical procedures, and the use of new radio and chemotherapeutic protocols, the 5-year survival rate for lung cancer patients is poor and remains less than 15% [1]. This underscores the desperate need for novel strategies for early detection, prevention, and treatment of this disease.

Peroxisome proliferator-activated receptors (PPARs) have recently emerged as potential targets for the development of safe and effective therapies for lung cancer [3]. PPARs are ligand-activated transcription factors belonging to the nuclear hormone receptor superfamily [4]. They were initially found to be involved in the control of energy homeostasis and cell differentiation, proliferation, apoptosis, and inflammation. This suggested a role for PPARs in several disorders such as diabetes, metabolic syndrome, and atherosclerosis [5]. Early research also linked PPARs to carcinogenesis and, to date, PPARs have been implicated in solid organ cancers like breast, ovary, prostate, bladder, gastric, and colon as well as in leukemias [3]. Similarly, several studies have identified PPARs in lung cancer cells. Few tantalizing studies in animal models of lung cancer showed that modulation of specific PPARs results in decreased tumor burden. Hence, many studies are underway to test the impact of targeting these receptors for therapeutic purposes.

## 2. PPARs ARE MEMBERS OF THE NUCLEAR RECEPTOR SUPERFAMILY

Nuclear receptors (NRs) are a superfamily of phylogenetically related proteins that are ligand-dependent transcriptional regulators. A total of 48 NR genes have been identified in the human genome [4]. They regulate a diverse range of normal physiological functions such as homeostasis, reproduction, development, differentiation, and metabolism [5]. In addition, ligand-independent actions of several members of the NR superfamily have also been reported, which may explain their complex range of effects [5]. The NR superfamily includes receptors for classical steroid hormones (estrogens, androgens, progesterone, glucocorticoids, mineralocorticoids, and vitamin D3), bile acids, retinoic acids, and thyroid hormones. In addition, a large number of receptors have been identified through sequence similarity to known receptors, but lacking identified natural ligands. The latter are referred to as nuclear orphan receptors and PPARs fall into this latter category.

**Figure 1:**
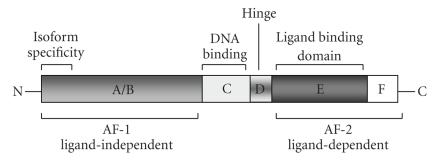
Structural organization of the functional domains of nuclear receptors.

Sequence alignment and phylogenetic tree construction resulted in in the classification of the NR family into six evolutionary groups of unequal size with PPARs in group 1 (NR1) along with thyroid and retinoic acid receptors [6]. All nuclear receptors share a common structural organization with multiple distinct functional domains (Figure 1). The N-terminal A/B domain contains at least one constitutively active transactivation region (AF-1) and several autonomous transactivation domains. The C domain is the most conserved region, responsible for DNA-binding specificity and essential for both homo- and heterodimerization of receptors. The D domain is a less conserved flexible hinge region between DNA-binding and the C-terminal ligand-binding domain E. The D domain contains the nuclear localization signal and also serves as docking site for cofactors. The E domain is a moderately conserved domain with a ligand-dependent transactivation function called AF-2. Some members also have a c-terminal F domain, whose sequence is extremely variable, and its structure and function are not known.

NR family members also share a common mode of action to regulate target gene expression. Ligand binding induces a conformational change in the receptor that permits homo- or heterodimerization, dissociation of corepressors, and concomitant association of coactivators. The homo- or heterodimer-coactivator complex binds to specific response elements in the promoter regions of target genes to regulate their transcription. Given the wide range of functions they regulate, it is not surprising that several members of the NR superfamily are implicated in various pathological conditions including the regulation of tumorigenesis. The effects of individual members are either beneficial or detrimental to tumorigenesis depending on the processes regulated by a given receptor and the tissue(s) in which it is expressed.

PPARs represent one of the intensively studied and well-characterized groups of NRs. Three subtypes of PPARs, encoded by three separate genes, have been identified and cloned: PPAR-*α* (NR1C1), PPAR-*β/δ* (NR1C2), and PPAR-*γ* (NR1C3) [6]. PPAR-*α* is the first member and was identified in the early 1990s in rodents as a receptor for compounds that induce peroxisome proliferation, which explains its name [7]. Subsequently, other two members were identified based on sequence similarity. Since then, PPARs have been recognized as important sensors for cellular fatty acids and fatty acid derivatives and mediate their effects through transcriptional regulation. Through these pathways, PPARs and their ligands are implicated in the regulation of cell proliferation, differentiation, and survival, and, therefore, carcinogenesis [8].

PPARs heterodimerize with retinoid X receptor (RXR) before binding a peroxisome proliferators response element (PPRE) in target genes. In addition to the induction of target gene expression, PPARs also mediate indirect repressive effects through transrepression by inhibiting the activity of key transcription factors via direct protein–protein interactions or by sequestrating cofactors necessary to their activity. In this fashion, PPAR-*α* and PPAR-*γ* interfere with NF-κB- and AP-1-mediated gene transcription, whereas PPAR-*β/δ* represses the expression of target genes induced by PPAR-*α* and PPAR-*γ* by binding to PPRE in association with corepressors [9–11].

Cofactors are proteins that can repress (corepressors) or enhance (coactivators) nuclear receptor transcriptional activity by bridging transcription factors to the basic transcription machinery or by specifically modifying chromatin structure. The nuclear receptor corepressor (NCoR), for example, and the silencing mediator of retinoid and thyroid receptors (SMRT) repress nuclear receptor activity. Their repressive effects are thought to occur through the recruitment of histone deacetylases (HDACs), but interactions with the basal transcriptional machinery might also play a role. The importance of corepressor interactions for PPAR-*α* and PPAR-*β/δ* action is currently poorly understood. The PPAR-*γ* interacting protein (PRIP/RAP250) and the PRIP-interacting protein with methyltransferase domain (PIMT) are two coactivators acting as molecular scaffolds which enhance PPAR-*γ* and RXR-mediated transcription. Importantly, the choice of PPAR/RXR heterodimers for PPAR target gene activation by PPAR agonists are related to the availability of cofactors such as CREB binding protein (CBP) and p300 versus SRC-1. Thus, the relative levels of cofactor expression control the specificity of the physiological response to PPAR or RXR agonists [12].

## 3. PPARs IN LUNG CANCER

In normal cells, the process of cellular differentiation is typically accompanied by cessation of proliferation, followed by senescence and, eventually, apoptosis. The balance between these events is disrupted in cancer cells. Therefore, the induction and maintenance of a differentiated state have been an important strategy in the search for cancer therapeutics [13]. The use of all-trans retinoic acid for the treatment of acute promyelocytic leukemia represents the first successful application of such an approach [14]. However, this approach has not been successfully exploited for the treatment of solid tumors. Since PPAR-*β/δ* and PPAR-*γ* play a key role in the differentiation of keratinocytes and adipocytes, it has been proposed that drugs capable of activating these receptors might be useful in arresting tumor growth [8, 15, 16]. In contrast, the role of PPAR-*α* in human carcinogenesis is less clear, but ligands that activate PPAR-*α* are implicated in the development of hepatocellular carcinoma in rodents [8, 17].

## 4. PPAR-*α*

PPAR-*α* is expressed in several tissues including liver, kidney, heart, skeletal muscle [18–20], vascular smooth muscle cells [21], endothelial cells [22], and monocytes/macrophages [23]. It was the first PPAR to be identified, and was shown to mediate peroxisome proliferators actions [18]. Peroxisome proliferators include several unrelated molecules such as steroids, lipids, hypolipidemic drugs (fibrates), industrial plasticizers, pesticides, and solvents that target the liver, among other organs, where they are known to induce peroxisome proliferation, liver hypertrophy, and hyperplasia, followed by hepatocellular carcinoma in rodents [18]. PPAR-*α* null mice are resistant to the effects of peroxisome proliferators (e.g., clofibrate) and PPAR-*α* ligands (e.g., Wy-14,643) as well as to the development of hepatocellular carcinoma in response to peroxisome proliferators [24]. The underlying mechanisms responsible for this effect remain incompletely understood. It has been proposed that peroxisome proliferators induce DNA replication and proliferation in hepatocytes in a PPAR-*α*-dependent manner [25, 26]. However, there is no direct evidence that PPAR-*α* effects the transcription of cell-cycle genes. Peroxisome proliferators are also reported to repress apoptosis in hepatocytes both in vitro and in vivo [27, 28]. The involvement of PPAR-*α* in this process was confirmed in studies using dominant negative PPAR-*α* in rat primary hepatocytes [29].

Interestingly, humans appear to be resistant to many of the adverse effects of the known peroxisome proliferators, but retain their beneficial effects. For example, epidemiological studies failed to show significant peroxisome proliferation in the liver of patients treated with hypolipidemic drugs [30, 31], and cell culture studies indicate that human cells display a reduced transcriptional response to PPAR-*α* activation when compared with rat cells [32]. These differences are important, but the mechanisms involved in their manifestation are unknown. Understanding the differences in the range of responses displayed by rodents and humans is one of the challenging aspects of PPAR-*α* biology. Today, very little is known about the role of PPAR-*α* in lung cancer biology and, thus, attention should be given to this area.

## 5. PPAR-*β/δ*

This PPAR isotype was first named as PPAR-*β* when isolated from Xenopus oocyte [33]. It was named PPAR-*δ* when it was subsequently identified in mouse [34], rat [35], and humans [36, 37], as it was not obviously homologous to the Xenopus gene. Nevertheless, it is now clear that both PPAR-*β* and *δ* are *bonafide* orthologues and, for clarity, it is referred to as PPAR-*β/δ*. The expression of PPAR-*β/δ* is broad since it has been detected in all of the tissues tested, with varied expression levels. It is expressed at relatively higher levels in the brain, adipose tissue, and skin [19, 38]. Several naturally occurring compounds such as saturated and polyunsaturated fatty acids and eicosanoids serve as PPAR-*β/δ* agonists in the micro molar range. However, similar to other PPARs, true physiological ligands of PPAR-*β/δ* are yet to be identified. Recently, synthetic agonists with affinities in the nanomolar range have been developed. GW501516 was the first synthetic PPAR-*β/δ* ligand developed by GlaxoSmithKline [39]. It was followed by Merck’s L-165,041 compound [40] and a 1,3,5-trisubstituted aryl compound by Novartis [41]. Unlike PPAR-*α* and PPAR-*γ* ligands, none of the PPAR-*β/δ* ligands are in clinical use, but they are in different stages of clinical testing.

The generation of receptor knock-out mice unveiled multiple developmental and homeostatic abnormalities in PPAR-*β/δ* null animals including placental defects, defects in myelination, decreased body fat, impaired wound healing, and altered inflammatory responses in skin [42–44]. Studies with high-affinity synthetic ligands revealed a critical role for PPAR-*β/δ* in glucose and lipid metabolism making it an important therapeutic target for the treatment of insulin resistance, glucose intolerance, hypertension and dyslipidemia (collectively known as metabolic syndrome or syndrome X), and with the potential to control weight gain, enhance physical endurance, improve insulin sensitivity, and ameliorate atherosclerosis [45].

Recent studies with knock-out mice and the treatment of human keratinocytes with high-affinity ligands have demonstrated that PPAR-*β/δ* plays a crucial role in the control of important cellular functions such as adhesion, proliferation, differentiation, and survival [8, 46]. Its role in lung cancer is less studied. However, in NSCLC cell lines, activation of PPAR-*β/δ* with GW501515 increased proliferation via stimulation of PI3-kinase/Akt signaling resulting in increased recognition of prostaglandin E_2_ via transcriptional upregulation of its EP4 receptor [47]. This contrasts PPAR-*β/δ* with PPAR-*γ* whose activation is consistently associated with inhibition of NSCLC proliferation.

## 6. PPAR-*γ*

PPAR-*γ* was discovered based on its similarity to PPAR-*α*, and it is the most intensively studied NR. By utilizing three different promoters, a single PPAR-*γ* gene encodes three isoforms namely PPAR-*γ*1, PPAR-*γ*2, and PPAR-*γ*3 [48]. Analysis of PPAR-*γ*1 and *γ*3 transcripts revealed that they both translate into the same PPAR-*γ*1 protein [49]. PPAR-*γ*2 protein contains an additional 30 amino acids at its N-terminus compared to PPAR-*γ*1. PPAR-*γ* is highly expressed in adipose tissue and it is a master regulator of adipocyte differentiation [50, 51]. In addition to its role in adipogenesis, PPAR-*γ* serves as an important transcriptional regulator of glucose and lipid metabolism, and it has been implicated in the regulation of insulin sensitivity, atherosclerosis, and inflammation [52–54]. PPAR-*γ* is also expressed in multiple other tissues such as breast, colon, lung, ovary, prostate, and thyroid where it was demonstrated to regulate cellular proliferation, differentiation, and apoptosis [55–58]. More recently, various leukocyte populations, including monocytes/macrophages, lymphocytes, and dendritic cells, have also been shown to express PPAR-*γ* suggesting a role for this molecule in the regulation of immune responses [59]. PPAR-*γ* has been described as a negative regulator of macrophage function since its activation suppresses the production of inflammatory cytokines, chemokines, metalloproteases, and nitric oxide [60, 61]. These PPAR-*γ* mediated anti-inflammatory effects are not restricted to monocytes, as treatment with PPAR-*γ* agonists results in inhibition of cytokine/chemokine production in several epithelial and stromal cell populations [62]. As will be discussed later, PPAR-*γ* activation also inhibits tumor progression in NSCLC [62, 63].

Since its discovery, several natural and synthetic compounds have been identified as activators of PPAR-*γ*. The insulin sensitizing antidiabetic drugs known as thiazolidinediones (TZDs) were the first compounds identified as PPAR-*γ* agonists [64]. The TZDs rosiglitazone and pioglitazone are currently in clinical use for the treatment of type-II diabetes, while troglitazone was withdrawn from clinical use because it was linked to idiosyncratic liver toxicity [65]. Other non-TZD synthetic ligands include certain nonsteroidal anti-inflammatory drugs such as isoxzolidinedione JTT-501 [66] and tyrosine-based GW7845 [67]. Naturally occurring compounds that activate PPAR-*γ* in vitro include polyunsaturated fatty acids, prostaglandin D_2_ (PGD_2_) and its metabolite 15-deoxy-Δ^12,14^ prostaglandin J_2_(15d-PGJ_2_), 12/15 lipoxygenase products 15-hydroxyeicosatetraenoic acid (15-HETE), and 13-hydroxyoctadecadienoic acid [68, 69]. However, none of these compounds activated PPAR-*γ* at physiologically relevant concentrations. More recently, intact nitroalkenes such as OA–NO_2_ (nitrated oleic acid) and LNO_2_ (nitrated linoleic acid) were observed to activate PPAR-*γ* at concentrations well within their detected levels in human plasma and urine making them ideal candidates for long-awaited endogenous ligands [70, 71]. It would be interesting to investigate whether nitroalkenes are present in tumor tissues, and their potential role in tumorigenesis. In addition, compounds from several medicinal plants such as Saurufuran A from * Saururus chinesis* [72], flavonoids such as chrysin and kampferol [73], phenolic compounds from *Glycyryhiza uralensis* [74], and curcumin from *Curcumin longa* [75, 76] are also shown to activate PPAR.

The synthetic ligands and some natural ligands have been used to elucidate the role of PPAR-*γ* in cellular functions both in vitro and in vivo. However, several caveats should be taken into consideration when interpreting such studies [3]. First, the natural ligands that regulate PPARs in vivo remain incompletely defined. Second, not all PPAR-*γ* ligands exert their effects through PPAR-*γ* since there is strong evidence for the activation of PPAR-*γ*-independent signals, particularly with the natural ligand 15d-PGJ_2_. Third, high-affinity ligands for PPAR-*γ* (e.g., the TZDs) may exert partial agonist/antagonist activity [77]. The latter might be due to the fact that individual TZDs induce different PPAR-*γ* conformations that influence the recruitment of different coactivator/corepressor molecules. Much information is now available regarding the potential role of PPAR-*γ* and its ligands in lung cancer and, thus, the rest of the discussion will focus on this topic.

## 7. PPAR-*γ* and PPAR-*γ* LIGANDS IN LUNG CANCER

PPAR-*γ* is expressed in many cancers including colon, breast, and prostate, and with few exceptions, PPAR-*γ* ligands are generally antiproliferative in these settings. Similarly, PPAR-*γ* is expressed in SCLC and NSCLC [78]. Furthermore, PPAR-*γ* ligands induce growth arrest and promote changes associated with differentiation as well as apoptosis in a variety of lung carcinoma cell lines, although most of the knowledge available in this area has been generated in NSCLC [3, 62]. The exact mechanisms linking modulation of PPAR-*γ* with cancer growth inhibition remain incompletely elucidated; however, strong evidence suggests that PPAR-*γ* ligands modulate the intracellular machinery involved in cell signaling and cell cycle control, and inhibit tumor cell recognition of extracellular mitogenic signals. Yet, other studies suggest that modulation of PPAR-*γ* affects the expression of angiogenic factors needed for the development of the vascular network responsible for supplying nutrients to tumor cells. These mechanisms are discussed below as they relate to the action of PPAR-*γ* ligands in lung cancer.

### 7.1. PPAR-*γ* ligands interfere with tumor cell signaling and cell-cycle control

Several observations point to targets for PPAR-*γ* ligands in the intracellular machinery responsible for cell-cycle control in tumor cells. For example, PPAR-*γ* ligands have been found to inhibit the growth of A549 adenocarcinoma cells due to G0/G1 cell cycle arrest through the upregulation of mitogen-activated protein kinases Erk1/2 and the downregulation of G1 cyclins D and E [62]. Troglitazone inhibits NSCLC proliferation in part by stimulating the expression of the GADD 153 (for growth arrest and DNA damage inducible gene-153) [79]. PPAR*γ* ligands can also trigger the activation of the mitogen-activated protein Kinase (MAPK) Erk cascade, which plays a central role in intracellular signaling by many extracellular stimuli. Interestingly, PPAR*γ* itself is a target for Erks, and Erk5 was reported to interact with PPAR-*γ*, but unlike the other MAPKs, this interaction induces activation rather than inhibition of PPAR-*γ* transcriptional activity [80]. Troglitazone was found to induce the apoptosis of NCI-H23 cells via a mitochondrial pathway through the activation of Erk1/2 [81]. In that study, the pro-apoptotic effects of troglitazone were clearly mediated via PPAR-*γ* since PPAR-*γ* siRNA blocked the response. Others have shown similar results using CRL-202 cells, and further demonstrated that troglitazone downregulated the expression of the pro-apoptotic molecules Bcl-w and Bcl-2, and decreased the activity of SAPK/JNK [82]. PPAR-*γ* ligands also induce the expression of death receptor 5 (DR5) and increase DR5 distribution at the cell surface in addition to reducing c-FLIP levels in human lung cancer cells. These agents cooperated with TRAIL to enhance apoptosis in human lung carcinoma cells [83].

Tumor suppressor genes are also affected by PPAR-*γ* ligands. For example, PGJ_2_ and ciglitazone stimulated the expression of p21 mRNA and protein expression in NSCLC, and this coincided with a reduction in cyclin D1 mRNA expression [84]. Of note, p21 antisense oligonucleotides significantly blocked lung carcinoma cell growth inhibition observed with PPAR-*γ* ligands thereby establishing an important role for p21 in this process. These findings are consistent with those of others showing that the proliferation of A549 cells injected subcutaneously into nude mice was inhibited significantly by treatment with ciglitazone, and this coincided with increased expression in tumors of PPAR-*γ* and p21, and with downregulation of cyclin D1 [85]. A connection between p53, another tumor suppressor gene, and PPAR-*γ* ligands has also been demonstrated by showing that 15-deoxy-PGJ_2_, together with docetaxel, stimulates apoptosis in NSCLC through inhibition of Bcl2 and cyclin D1, and overexpression of caspases and p53 [86].

**Figure 2:**
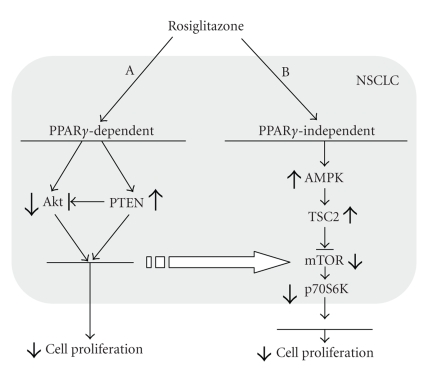
Rosilitazone stimulates NSCLC proliferation by affecting the Akt/mTOR pathway through PPAR*γ*-dependent and PPAR*γ*-independent mechanisms.

Recent reports implicate alterations in the mammalian target of rapamycin (mTOR) signaling pathway in the antitumor effects of PPAR*γ* ligands. Rosiglitazone, for example, was reported to reduce the phosphorylation of Akt, an upstream positive modulator of mTOR, and increase PTEN, a negative modulator of mTOR, in NSCLC H1792 and H1838 cells; this resulted in inhibition of cell proliferation [87] (Figure 2). Although the effects of rosiglitazone on Akt and PTEN were blocked by the selective PPAR-*γ* antagonist GW9662 and restored by transient overexpression of PPAR-*γ*, cell growth was not entirely restored suggesting the involvement of additional PPAR-*γ*-independent mechanisms of action. These observations are consistent with the work of others showing similar increases in PTEN expression induced by rosiglitazone [88]. Further work revealed that rosiglitazone increased the phosphorylation of AMPK*α*, a target of LKB1 and upstream downregulator of mTOR [87]. Rosiglitazone may also activate TSC2, another potential tumor suppressor and upstream downregulator of mTOR. The latter pathway was independent of PPAR-*γ* since it was not affected by GW9662 or PPAR-*γ* siRNA. This again highlights the fact that TZDs may act via PPAR*γ*-independent pathways. This is important since TZDs display proinflammatory activities in part via their ability to augment PPAR-*β/δ* signaling. Thus, some effects of PPAR-*γ* ligands may be mediated through an off-target effect [89]. These studies emphasize the need for PPAR modulators with increased receptor subtype specificity.

### 7.2. PPAR-*γ* ligands inhibit tumor cell recognition of extracellular mitogenic factors

Several studies suggest that PPAR-*γ* ligands exert their antitumor effects by blocking access to mitogenic agents such as PGE_2_, a major cyclooxygenase metabolite that plays important roles in tumor biology. The functions of PGE_2_ are mediated through one or more of its receptors EP1, EP2, EP3, and EP4 [90]. Human NSCLC cell lines express EP2 receptors, among other EP receptors, and the inhibition of cell growth by PPAR-*γ* ligands like GW1929, PGJ_2_, ciglitazone, troglitazone, and rosiglitazone is associated with a significant decrease in EP2 mRNA and protein expression. Notably, the inhibitory effects of rosiglitazone and ciglitazone, but not PGJ_2_, were reversed by a specific PPAR-*γ* antagonist GW9662, suggesting the involvement of PPAR-*γ*-dependent and PPAR-*γ*-independent mechanisms [90].

Other studies suggest that PPAR-*γ* ligands might prevent the interaction of tumor cells with their surrounding stroma, thereby interfering with host-derived and tumor-derived factors with mitogenic and prosurvival effects. An example of this is fibronectin, a matrix glycoprotein that resides in the lung stroma that is increased in most, if not all, chronic forms of lung disease [91]. This is true for tobacco-related lung disorders and fibrotic disorders, all associated with increased incidence of lung cancer [92]. Several studies suggest that fibronectin serves as a mitogen and survival factor for NSCLC [93], and fibronectin was recently shown to stimulate tumor cell expression of matrix metalloproteinases, proteases implicated in metastatic disease [94]. These observations support the idea that tumor cell interactions with fibronectin through surface integrin receptors are advantageous for tumors since they stimulate proliferation, survival, and metastases [93]. This idea remains to be proven in vivo, but if found to be true, this might unveil a new target for anticancer strategies. In this regard, PPAR-*γ* ligands were shown to inhibit fibronectin expression in NSCLC cells by inhibiting transcription factors involved in regulation of fibronectin gene expression [95]. PPAR-*γ* ligands (rosiglitazone and GW1929, but not PGJ_2_) were also recently reported to inhibit the expression of the gene encoding for the *α*5 integrin subunit resulting in reduced expression of the integrin *α*5*β*1, a fibronectin receptor that mediates fibronectin’s mitogenic effects in NSCLC cells and nontumor lung cells [96]. Thus, by inhibiting the expression of fibronectin and its integrin *α*5*β*1, PPAR-*γ* ligands might reduce tumor cell recognition of fibronectin with consequent changes in cell proliferation and apoptosis.

### 7.3. PPAR-*γ* ligands inhibit angiogenesis and tumor vascularization

The idea that PPAR-*γ* might regulate the generation of the complex vascular network that supplies tumor cells is supported by studies showing significant reduction in blood vessel density in the lung tumors generated by the injection of A549 cells into the flanks of SCID mice treated with PPAR*γ* ligands [97]. In studies in vitro, the treatment of A549 cells with troglitazone or their transient transfection with a constitutively active PPAR-*γ* construct blocked the production of angiogenic molecules such as ELR+CXC chemokines IL-8 (CXC-8), ENA-78 (CXCL5), and Gro-alpha (CXCL1) [97]. Moreover, conditioned media from untreated A549 cells stimulated human microvascular endothelial cell chemotaxis, whereas the condition media of troglitazone-treated A549 was inhibitory. Of note, PPAR*γ* activation inhibited NF-κB, a transcription factor known to regulate the expression of many of the pro-angiogenic factors mentioned above. Similarly, rosiglitazone was shown to inhibit mouse lung tumor cell growth and metastasis in vivo through direct and indirect anti-angiogenic effects [63].

### 7.4. PPAR-*γ* is a novel candidate for targeting tumor microenvironment

In tumors, cancer cells coexist with different cell types including fibroblasts, macrophages, endothelial cells, and multitude of diverse cytokines and chemokines secreted by these cells, constituting a distinct tumor microenvironment. One of the important conceptual advances in tumor biology in recent years has been the appreciation that all major aspects of a cancer cell are influenced by the tumor microenvironment. Interestingly, PPAR-*γ* is expressed in all major cell types present in the tumor microenvironment, and its ligands have been shown to inhibit several of the pro-tumorigenic functions of these cell types in vitro and, in some cases, in vivo. For example, PPAR-*γ* ligands were shown to inhibit proliferation, and induce apoptosis, migration, and tube formation in endothelial cells [98]. Also, PPAR-*γ* ligands can inhibit the transdifferentiation of fibroblasts into myofibroblasts, a phenotype similar to that of tumor-associated fibroblasts, in several fibrotic conditions [99–102]. A recent study demonstrated that PPAR-*γ* ligands completely reverse the antitumor cytotoxic T-lymphocyte suppressive activity and the M2 phenotype of tumor-associated macrophages [103]. PPAR-*γ* ligands are also known to inhibit the expression of several cytokines and chemokines produced by all of the major cell types present in the tumor microenvironment (60, 61, 97, 98]. Together with data showing effects on fibronectin matrix expression and recognition in NSCLC [95], the above observations suggest that PPAR-*γ* might be a novel candidate for targeting the tumor microenvironment.

## 8. IMPLICATIONS FOR THERAPY AND RESEARCH NEEDS

The studies mentioned above suggest that PPARs are involved in lung cancer cell biology. However, their roles remain uncertain, and much needs to be learned before they are targeted for therapeutic intervention, especially when considering PPAR-*α* and PPAR-*β/δ*. Activation of PPAR-*γ* is strongly associated with decreased lung carcinoma cell proliferation both in vitro and in vivo. Furthermore, in primary NSCLC, the expression of PPAR-*γ* has been correlated with tumor histological type and grade, and decreased PPAR-*γ* expression was correlated with poor prognosis [104]. Because of this, and the fact that synthetic agonists of PPAR-*γ* with good safety profiles are currently in use in the clinical arena, PPAR-*γ* has emerged as a reasonable target for the development of anti-lung cancer therapies. Synthetic and natural PPAR-*γ* activators might be useful. For example, arachidonic acid treatment inhibits the growth of A549 cells, and this effect is blocked by the synthetic PPAR-*γ* inhibitor GW9662 [105]. MK886, a 5-lipoxygenase activating protein-directed inhibitor, stimulates apoptosis and reduces the growth of A549 cells through activation of PPAR*γ* [106]. These and related drugs can be used alone or in combination with other drugs for synergistic effects. This was observed when using low doses of MK886 in combination with ciglitazone and 13-cis-retinoic acid on A549 and H1299 cells [106]. Also, dramatic synergistic anticancer effects have been reported for lovastatin (an HMG-CoA reductase inhibitor) and the PPAR-*γ* ligand troglitazone in several cell lines including lung cancer cells [107]. An enhancement by rosiglitazone of the antitumor effects of gefitinib on A549 cell growth was recently noted suggesting that combination strategies using selective nuclear receptor activators in conjunction with epidermal growth factor receptor inhibitors might prove effective [108].

Although little information is available in vivo, emerging data are beginning to unveil potential implications to the human condition. In this regard, a retrospective analysis of a cohort of 87 678 individuals identified through the Veterans Integrated Services Network 16 data warehouse revealed a 33% reduction in lung cancer risk among TZD users compared with nonusers after adjusting for confounder variables. Interestingly, a similar risk reduction was not observed for colorectal and prostate cancers [109].

Despite the above, enthusiasm for this approach should be tempered by work showing that the PPAR-*γ* ligands rosiglitazone, ciglitazone, and PGJ_2_ were found to stimulate PPAR-*γ* transactivation in lung adenocarcinoma cell lines in vitro, but little to no effects were noted in squamous cell or large cell carcinomas suggesting that their anticancer properties might not be shared by all lung tumors, or that important PPAR-*γ*-independent pathways are at play [108, 110]. Thus, a better understanding of the mechanisms of action of activated PPARs in tumors (and host cells) is required since the dissection of these pathways might unveil better targets for therapy. Nevertheless, the data available to date regarding PPAR-*γ* is promising and justify engaging in prospective, randomized, clinical studies to determine the true role of PPAR-*γ* ligands in lung cancer, while further work is performed to identify more selective and effective strategies.
